# PD-1 and LAG-3 expression in EBV-associated pediatric Hodgkin lymphoma has influence on survival

**DOI:** 10.3389/fonc.2022.957208

**Published:** 2022-08-05

**Authors:** Oscar Jimenez, Tamara Mangiaterra, Sandra Colli, Mercedes García Lombardi, Maria Victoria Preciado, Elena De Matteo, Paola Chabay

**Affiliations:** ^1^ Multidisciplinary Institute for Investigation in Pediatric Pathologies (IMIPP), CONICET-GCBA, Molecular Biology Laboratory, Pathology Division, Ricardo Gutiérrez Children’s Hospital, Buenos Aires, Argentina; ^2^ Pathology Division, Ricardo Gutiérrez Children’s Hospital, Buenos Aires, Argentina; ^3^ Oncology Division, Ricardo Gutiérrez Children’s Hospital, Buenos Aires, Argentina

**Keywords:** hodgkin lymphoma, tolerogenic, PD-1, LAG-3, pediatric

## Abstract

In pediatric Hodgkin lymphoma (HL), the inability of the cytotoxic microenvironment induced by EBV presence to eliminate tumor cells could reflect the fact that the virus might be able to induce the expression of exhaustion markers to evade an immune response. Therefore, the expression of exhaustion markers in pediatric EBV–associated HL was evaluated. A balance between cytotoxic GrB and Th1 Tbet markers with regulatory Foxp3 was proved in EBV+ cases. In addition, exclusively in EBV-associated cHL, a correlation between PD-1 and LAG-3 expression was observed. Furthermore, those cases also displayed a trend to worse survival when they expressed LAG-3 and inferior event-free survival when both PD-1 and LAG-3 molecules were present. Therefore, even though a cytotoxic and inflammatory environment was supposed to be triggered by EBV presence in pediatric cHL, it seems that the virus may also induce the synergic effect of inhibitory molecules LAG-3 and PD-1 in this series. These observations may reflect the fact that the permissive and exhausted immune microenvironment succeeds to induce lymphomagenesis.

## Introduction

Classic Hodgkin lymphoma (cHL) is unique among lymphoid malignancies due to the chemokine and cytokine production by Hodgkin–Reed–Sternberg (HRS) tumor cells, which, in turn, delineates a complex microenvironment of non-tumor cells. The microenvironment consists of both innate and adaptive immune cells (1).

The microenvironment in cHL is also modulated by the presence of the Epstein–Barr virus (EBV) (2). In adult patients with cHL, the presence of Tregs is more pronounced in the microenvironment of EBV+ cases, along with CD4+ Th2 cells, CD56+ NK (Natural Killer) cells, and cytotoxic CD8+ T cells expressing GrB and TIA (3). In pediatric cHL, EBV+ cases displayed a cytotoxic/Th1 profile, characterized by higher numbers of CD3+, CD8+, TIA1+, and TBET+ lymphocytes (4).

In our pediatric population, EBV-associated lymphomas are significantly increased in children younger than 10 years (5). In both EBV+ diffuse large B-cell lymphoma (DLBCL) and cHL, viral presence is associated with an increase in cytotoxic GrB+ cells (6). However, cytotoxic cells may be unable to eliminate tumor cells since persistent antigenic stimulation leads to T-cell exhaustion, which expresses high levels of inhibitory receptors, including programmed cell death protein1 (PD-1) and lymphocyte activation gene 3 protein (LAG-3) (7). Exhausted T cells express high levels of inhibitory receptors and produce less effector cytokines and lose the ability to eliminate cancer (7). Therapeutic immune checkpoint blockade has shown outstanding efficacy in restoring effector T-cell function at the microenvironment (8).

In cHL, HRS cells exhibit a high expression of PD-1 ligands (PDL-1) on their surface, but PDL-1 expression was also described on tumor-associated macrophages. PDL-1 in both malignant and non-malignant cells engage PD-1+ tumor-infiltrating T cells, drive their exhaustion, and, as a result, inhibit the immune responses in cHL (8). PD-1 includes both an immunoreceptor tyrosine-based switch motif (ITSM), essential for the transmission of inhibitory signals, and an immunoreceptor tyrosine-based inhibitory motif (ITIM). After ligation with its ligands, both PD-1 motives become phosphorylated, which consequently leads to the depression of various intracellular signaling pathways (9). CD4+ T lymphocytes, including PD-1+ ones, are enriched in proximity to HRS cells, forming rosettes in a fraction of cHL samples (10). Increased PD-1+ tumor-infiltrating lymphocytes (TILs) have been associated with a poorer prognosis in cHL patients (11). LAG-3 is expressed by several cell types, like T, NK, B, and dendritic cells, and it interacts with MHC class II, playing a negative regulatory role and suppressing T-cell function (12). LAG-3 expression is frequently found in the surrounding immune infiltrating cells. They seem to be higher in regions adjacent to the malignant cell, have Treg-like features (13), and interact essentially with MHC-II expressed by tumor- or antigen-presenting cells to trigger inhibitory signaling that suppresses T-cell function (14). Furthermore, LAG-3 acts synergistically with PD-1 and/or CTLA-4 to negatively regulate T-cell expansion (15). EBV latency III–transformed B cells exhibit strong immunoregulatory properties since they induce regulatory T cells that express PD-1 (16). In EBV-associated adult and pediatric cHL, PD-1 expression was not related to viral presence (17), whereas LAG-3 expression remains unexplored so far.

In Argentina, the cytotoxic environment was also proved in EBV+ pediatric cHL (6), which could be counterbalanced by PDL-1 cells at the microenvironment (18). Given that checkpoint blockade therapy may have relevance in a population with increased pediatric EBV-associated lymphomas (5), the characterization of the cHL microenvironment might provide relevant information on the response to checkpoint blockade therapy. Therefore, the aim of this study is to further describe the tolerogenic environment in EBV-associated childhood cHL to establish whether this population could be targeted by this specific therapy.

## Methods

### Patients and samples

Formalin-fixed paraffin-embedded (FFPE) biopsy samples from 35 patients were collected retrospectively, on the basis of the availability of sufficient material, from the archives at the Pathology Division, Ricardo Gutierrez Children’s Hospital in Buenos Aires, Argentina, from 1990 to 2012. The age range was 2–18 years (median: 9.5 years). The sample was obtained at diagnosis before treatment. The treatment followed the Grupo Argentino de Tratamiento de la Leucemia Aguda (GATLA) protocol.

Institutional guidelines regarding human experimentation were followed, and they were in accordance to the Helsinki Declaration of 1975.The Ricardo Gutierrez Children Hospital Ethics Committee approved the study, and all the patients’ guardians gave informed consent for the study.

### EBER *in situ* hybridization

*In situ* hybridization for EBERs (Epstein Barr virus encoded RNAs) was performed in FFPE tissue sections and assessed using labeled oligonucleotides with fluorescein isothiocyanate (FITC) as probes (Ok) according to the manufacturer’s instructions. An anti-FITC monoclonal antibody labeled with alkaline phosphatase was used to detect hybridized sites.

### Immunohistochemistry

Immunohistochemistry (IHC) for CD4, CD8, Foxp3 (Tregs), GrB (cytotoxic cells), and PD-1 was performed on FFPE samples as described (6, 18). In addition, immunohistochemical staining with primary antibodies for Tbet (Th1 cells) (Santa Cruz Biotechnology, Texas, USA), CD56 (NK cells) (Abcam), and LAG-3 (Abcam, Cambridge, UK) was performed to extend previous studies (6, 18). Primary antibodies were detected using the Vectastain-ABC-Peroxidase kit, using diaminobenzidine (DAB) as a chromogen. The numbers of labeled cells were determined using the image analysis free software Image J. The cells were counted optically without the use of a plug-in. The results were expressed as positive cells per area unit (cells+/mm^2^).

Viral LMP1 (latent membrane protein 1) expression in EBERs+ tumor cells was assessed with monoclonal antibodies CS1-4 (Dako). IHC detection of the primary antibody was carried out using a universal streptavidin–biotin–complex peroxidase detection system (UltraTek HRP Anti-Polyvalent Lab Pack, ScyTek, Utah, USA), according to the manufacturer’s instructions.

### Statistical analysis

Statistical analysis was performed using GraphPad Prism5 (GraphPad Software Inc, San Diego, CA, USA). Categorical variables were analyzed using Fisher’s exact test. Mann–Whitney test was used to compare the means between groups in relation to EBV presence. Correlations between data were determined using Spearman correlation test. Follow-up for survival was available in 23 patients. For survival analyses related to PD-1 and LAG-3, expression at the microenvironment was considered as positive and negative above and below 1% of positive cells (positive cells/total cells ×100) for each marker as cutoff, respectively. Kaplan–Meier curves based on the abovementioned cutoff thresholds were generated, and the statistical significance of each marker was determined using the log-rank test. All tests were two-tailed, and p < 0.05 was considered statistically significant.

## Results

**Figure 1:**
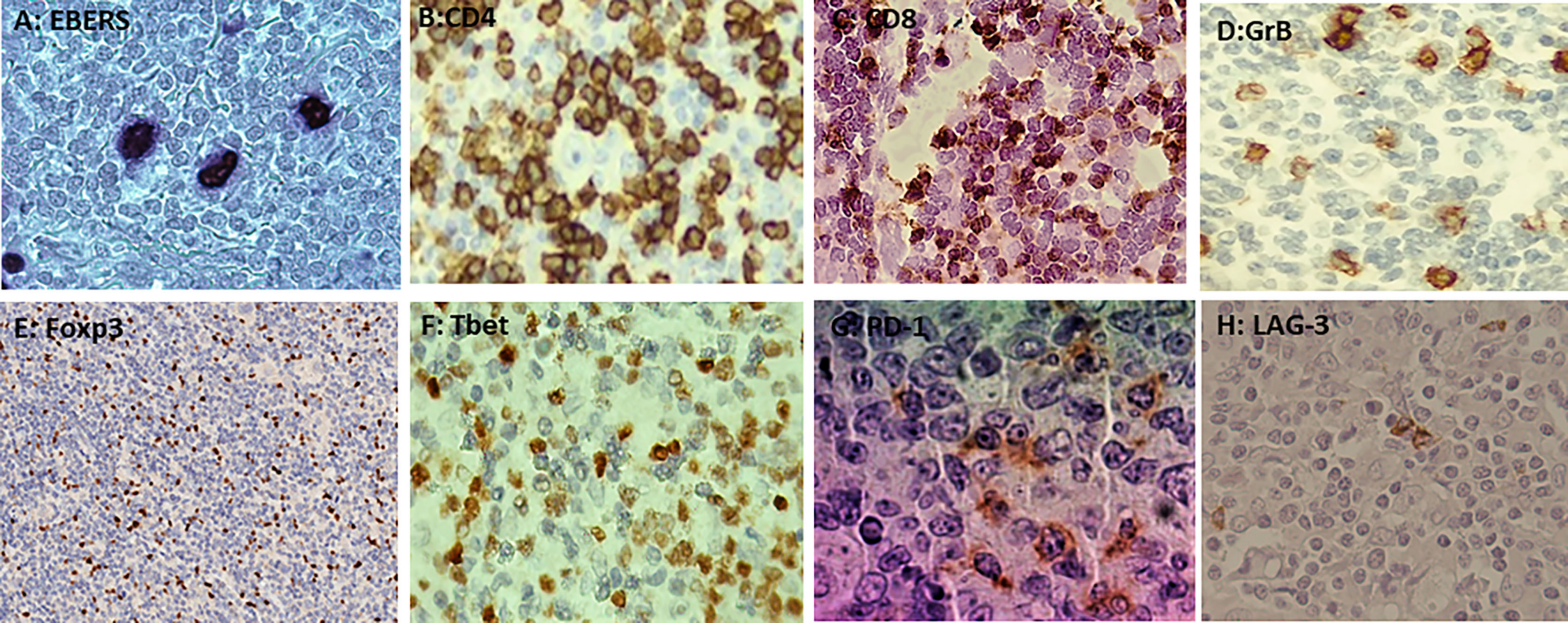
**(A)** Nuclear staining of EBERs in Hodgkin–Reed–Sternberg by ISH (*in situ* hibridization). **(B)** Membranous staining of CD4 in the microenvironment by immunohistochemistry (IHC). **(C)** Membranous staining of CD8 in the microenvironment by IHC. **(D)** Cytoplasmatic granular staining of GrB in the microenvironment by IHC. **(E)** Nuclear staining of Foxp3 in the microenvironment by IHC. **(F)** Membranous and cytoplasmic staining of Tbet in the microenvironment by IHC. **(G)** Membranous and cytoplasmic staining of PD-1 in the microenvironment by IHC. **(H)** Membranous and cytoplasmic staining of LAG-3 in the microenvironment by IHC.

Both EBERs and LMP1 expression were observed in 74% (26/35) pediatric cHL cases (**Figure 1**, **Supplementary Table 1**). No differences between EBV+ and EBV- cases were observed in CD4, CD8, Foxp3, GrB, and Tbet mean cell counts (p>0.05, Mann–Whitney test) (**Figure 1**, **Supplementary Figure 1**) Even though it was described in children with cHL from Brazil that EBV presence in pediatric cHL may trigger a cytotoxic and inflammatory environment (4), in the current series, this environment could be counterbalanced by a regulatory milieu, since in EBV+ cases, a statistical positive correlation between Foxp3+ cells as a marker of Tregs, with GrB+ ones, as a marker of cytotoxicity (r=0.706, p<0.0001), was observed. Furthermore, a statistical positive correlation between Foxp3 and Tbet, as a marker of Th1 (r=0.536, p=0.002), was also proved. Those findings were not observed in EBV- cHL cases.

LAG-3 expression at the microenvironment was observed in 57% (20/35) of the cases (**Figure 1**); 70% (14/20) of LAG-3+ cases were also EBV+, but no statistical difference was observed in the LAG-3+ mean cell count when EBV+ cases were compared with EBV- ones (p>0.05) (**Supplementary Figure 1**). Concerning PD-1 expression, it was proved in 83% (29/35) of the cases (**Figure 1**); 69% (20/29) were also EBV+ but not statistically associated with PD-1 presence (p>0.05). In addition, mean PD-1+ cells in EBV+ cases were no statistically different from EBV- ones (p>0.05) (**Supplementary Figure 1**).

With the purpose of investigating PD-1 and LAG-3 expression at the microenvironment with other TIL markers, correlation analysis was performed. In the whole series, the LAG-3+ cell count displayed a statistical positive correlation only with CD8+ cells (r=0.374, p=0.034). In line with this, the PD-1+ cell number also showed a trend to a positive correlation with CD8+ cells (r=0.311, p=0.054), while no correlation was observed with the remaining markers (p>0.05). Remarkably, LAG-3+ cells showed a statistical positive correlation with PD-1+ cells (r=0.388, p=0.028) in all pediatric cHL cases. To further explore the influence of EBV presence, when this correlation was evaluated exclusively in EBV+ cases, it remained significant (r=0.518, p=0.011), but it was lost in EBV- ones (p>0.05) (**Supplementary Figure 2**). Exclusively in EBV- pediatric cHL, a statistically positive correlation was demonstrated between LAG-3+ and Foxp3+ cell counts (r=0.828, p=0.004) and a trend between the former and CD8+ cells (r=0.663, p=0.083).

**Figure 2:**
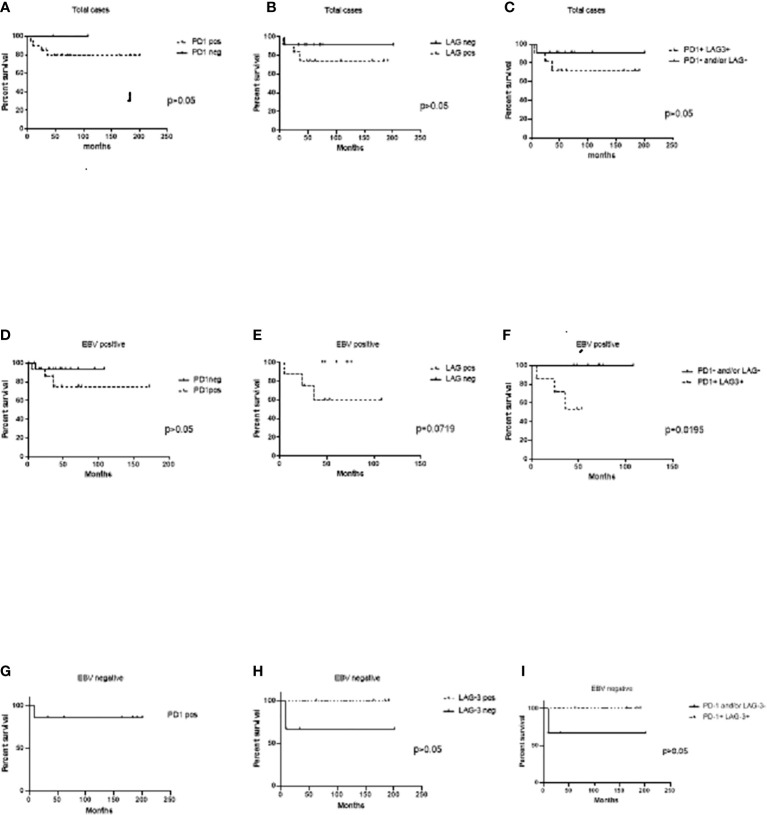
Event-free survival (EFS) analysis to PD-1 and LAG-3 expression at the microenvironment. **(A)** Cases expressing PD-1+cells at the microenvironment versus PD-1- ones in the whole series. **(B)** Cases expressing LAG-3+ at the microenvironment versus LAG-3-ones in the whole series. **(C)** Cases expressing both PD-1+/LAG-3+ cells at the microenvironment versus PD-1- and/or LAG-3 ones in the whole series. **(D)** EBV+ cases expressing PD-1+cells at the microenvironment versus PD-1- ones. **(E)** EBV+ cases expressing LAG-3+ at the microenvironment versus LAG-3- ones. **(F)** EBV+ cases expressing both PD-1+/LAG-3+ cells at the microenvironment versus PD-1- and/or LAG-3 ones. **(G)** EBV- cases expressing PD-1+ cells at the microenvironment versus PD-1- ones. **(H)** EBV- cases expressing LAG-3+ at the microenvironment versus LAG-3- ones. **(I)** EBV- cases expressing both PD-1+/LAG-3+ cells at the microenvironment versus PD-1- and/or LAG-3 ones.

Survival analysis to evaluate LAG-3 and PD-1 influence was performed. In the whole series, neither LAG-3 nor PD-1-positive expression was associated with worse survival (p>0.05) (**Figures 2A, B**). Furthermore, when cases expressing both PD-1 and LAG-3 markers in the entire series were evaluated, no influence on survival was also evidenced (p>0.05) (**Figure 2**). Given that it seems that LAG-3 and PD-1 were statistically correlated in EBV+ cases, individual as well as combined expression was investigated in EBV-associated cases. Even though cases positive for PD-1 did not exhibit differences in survival in relation to their negative counterparts (p>0.05) (**Figure 2**), LAG-3 expression showed a trend to worse survival in EBV-associated pediatric cHL, since 5-year survival was 60% in LAG-3+ versus 100% in LAG-3- ones (p=0.0719) (**Figure 2**). Remarkably, in EBV+ cases with LAG-3 and PD-1 coexpression, 5-year survival was 54% in comparison with the 100% observed in LAG-3-/PD-1-, LAG-3+/PD-1-, or LAG-3-/PD-1+ cases. Therefore, LAG-3+/PD-1+ expression was associated with worse survival exclusively in EBV-associated pediatric cHL (p=0.0195) (**Figure 2**). Neither PD-1 nor LAG-3 alone or combined expressions had influence on survival in EBV-negative cases (**Figure 2**).

## Discussion

Barros et al. previously characterized in EBV+ pediatric cHL a more intense T-cell infiltrate, with higher numbers of CD3+, CD8+, TIA1+, and TBET+ lymphocytes (4). However, in the context of persistent viral infection, persistent stimulation could induce T-cell functional exhaustion. Even though a cytotoxic M1 polarized milieu was proved (6), our group also described an increase in PDL-1+ cells exclusively at the microenvironment in EBV-associated cHL (18), which prompted us to further explore EBV involvement in the process of exhaustion to restrain the viral-induced cytotoxicity. The correlation between Foxp3+ regulatory T cells with both GrB+ and Tbet+ ones, as a marker of cytotoxic and Th1 environments, respectively, in EBV+ cases demonstrates this delicate balance, which seems to be in favor of regulation, given the fact that the lymphomagenesis process progresses even in a cytotoxic as well as proinflammatory milieu.

Concerning immune exhaustion, PD-1 expression was markedly upregulated on tumor-infiltrating CD8+ T cells and correlated with reduced cytokines in Hodgkin’s lymphoma, melanoma, hepatocellular carcinoma, and gastric cancer patients (7). PD-1 engagement triggers a signaling cascade that results in TCR signal attenuation that inhibits T-cell activation, proliferation, and cytokine production. Antigen persistence leads to ongoing PD-1 expression and eventual T-cell exhaustion (19). In EBV+ DLBCL patients, the expression of a few reactive PD1+ TILs was described (20). In line with this, it was suggested that viral LMP1 oncoprotein may sustain an immunosuppressive microenvironment by the induction of immunosuppressive cytokines and the expression of PD-1 (21). In contrast, in our pediatric cHL, EBV presence may not influence PD-1 expression in TILs.

LAG-3 is expressed on CD4+ and CD8+ T cells, CD4+Foxp3+ Treg, B cells, plasmacytoid dendritic cells, and NK cells, and binds to MHC class II, and other ligands such as the known ligands that include galectin-3, LSECtin, alpha-synuclein fibers, and FGL-1 (22). It was highly expressed on CD4+ or CD8+ T cells with reduced function at the microenvironment in adult follicular lymphoma (FL) (23). In line with this, a remarkable expression of LAG-3 was observed in adult cHL, in the proximity of HRS cells (24), expressed by Tregs (25). In our series, LAG-3 might tend to be expressed by CD8+ T cells since a significant positive correlation was observed between both markers. However, in EBV- cases, LAG-3 could be expressed by regulatory Foxp3+ cells based on their statistical correlation. Recently, high LAG-3 expression was also demonstrated in pediatric cHL from a developed population (26). In contrast, LAG-3 expression in our series seems to be lower than that previously described for pediatric and adult cHL, most of them skewed to EBV-associated cases.

It has recently been shown that LAG-3 synergistically impacts T-cell function with PD-1. Indeed, LAG-3 was coexpressed with PD-1 and almost exclusively expressed on intratumoral PD-1+ T cells in FL (23). In human ovarian cancer, LAG-3+PD-1+CD8+T cells were more dysfunctional in IFN-γ and TNF-α production compared with LAG-3+PD-1- or LAG-3-PD-1-CD8+subsets (7). The joint expression of both exhaustion markers was also related to resistance to PD-1 blockade therapies in a mouse model of lung adenocarcinoma (27). Furthermore, PD-1+LAG-3+TILs exhibited a more exhausted phenotype and function than single positive or negative TILs; the dual blockade of PD-1 and LAG-3 resulted in tumor regression (7). The expression of LAG-3 and PD-1 at the microenvironment was also proved by our study, as suggested by the correlation of both markers. Moreover, previous studies reported in chronic viral infections the coexpression of LAG-3 with PD-1 on T cells as a contribution to the development of exhaustion (28). Of note, the correlation between LAG-3 and PD-1 observed in EBV-associated pediatric cHL, not proved on the non-associated cases, supports the notion of viral infection to promote exhaustion. Even though an increased LAG-3 expression was demonstrated previously in EBV-associated adult cHL, related to the loss of viral LMP-1 specific T-cell function (16), it was not proved in relation to PD-1 expression as observed in our series.

Increased PD-1+ TILs have been associated with a poorer prognosis in adult cHL patients (11), whereas cases positive for triple positive (EBV+, HRS-PD-L1+, PD-1+ TILs) identified high-risk cHL patients (29). In our series, PD-1 expression alone does not seem to be involved in survival in pediatric cHL. In addition, no association between the expression of LAG-3 and clinical parameters or outcome was also demonstrated in adult cHL cases (25). In contrast, in adult patients with FL, LAG-3 expression on intratumoral T cells correlated with a worse outcome (23). Remarkably, the only report that revealed LAG-3 expression in pediatric cHL exhibited worse event-free survival (EFS) but in patients with the lowest density of LAG-3 expression in contrast to those with the highest density of expression that exhibited better survival (26). In line with this, our pediatric cHL cases displayed a trend to worse survival when LAG-3 expression was proved, even in cases with a low percentage of positive cells, but only in EBV-associated cases. Furthermore, significantly worse EFS was confirmed when both LAG-3 and PD-1 markers were expressed in EBV+ cases. These findings may exhibit a cooperative involvement between EBV on one hand and the synergistic effect of PD-1 and LAG-3 exhaustion molecules on the other to promote lymphomagenesis and possibly counteract the cytotoxic environment previously described in EBV-associated pediatric cHL (4, 6).

LAG-3 was proposed as a candidate for combination therapy with PD-1 blockade in adult cHL to restore T-cell function more efficiently than either one alone (30), whereas in pediatric patients, the scenario is quite unexplored. Even though a cytotoxic and inflammatory environment was supposed to be triggered by EBV presence in pediatric cHL (4, 6), it seems that the virus may also induce the expression of inhibitory molecules such as PDL-1 (18), along with the synergy effect of LAG-3 and PD-1 molecules observed in this series. These observations may reflect the fact that the permissive and exhausted immune microenvironment succeeds to induce lymphomagenesis.

## Data availability statement

The original contributions presented in the study are included in the article/**Supplementary Material**. Further inquiries can be directed to the corresponding author.

## Ethics statement

The studies involving human participants were reviewed and approved by Comite de ética en investigación, HNRG. Written informed consent to participate in this study was provided by the participants’ legal guardian/next of kin.

## Author contributions

PC conceived of the study and its design, drafted the manuscript and acquired funding. OJ and TM performed the experiments, the analysis and interpretation of data. MGL, EDM and MVP participated in its coordination and modification. All authors read and approved the final manuscript.

## Funding

This study was supported in part by grants from the National Agency for Science and Technology Promotion (PID clinic n°048 and 052- PICT 2017 1554-PICT 2018 0966) and Conicet (PUE 0058). CP, DME, and PMV are members of the CONICET Research Career Program. JO and MT are CONICET doctoral fellows.

## Acknowledgments

The authors thank Barbara Cao; Silvana Romero; Cristina Pabes and Maria Jose Andrade (Histopathological Laboratory, at the Ricardo Gutierrez Children’s Hospital) for their helpful histotechnical work.

## Conflict of interest

The authors declare that the research was conducted in the absence of any commercial or financial relationships that could be construed as a potential conflict of interest.

## Publisher’s note

All claims expressed in this article are solely those of the authors and do not necessarily represent those of their affiliated organizations, or those of the publisher, the editors and the reviewers. Any product that may be evaluated in this article, or claim that may be made by its manufacturer, is not guaranteed or endorsed by the publisher.

## Supplementary material

Supplementary Figure 1 Comparison of mean cell count between EBV+ and EBV- cases for **(A)** CD8, **(B)** CD4, **(C)** Foxp3, **(D)** GrB, **(E)** Tbet, **(F)** PD-1, **(G)** LAG-3.

Supplementary Figure 2 Correlation analysis for LAG-3 with CD8 in total cases, and between PD-1 and LAG-3 in total cases, EBV+ and EBV- cases.
