# Supplementary material for: PD-1 and LAG-3 expression in EBV-associated pediatric Hodgkin lymphoma has influence on survival
Source: Front Oncol. 2022 Aug 5;12:957208. doi: 10.3389/fonc.2022.957208 (PMC9390066; doi:10.3389/fonc.2022.957208)
Supplement: Supplementary file 1 [file DataSheet_1.pdf]

Table S1: General characteristic of included patients.

Variables	***Total***	***EBV+***	***EBV-***
Age *(median, range)*	9 (4-15)	8 (4-15)	11 (5-14)
Gender			
Male	34	26	8
Female	1	0	1
Histological Subtype			
Nodular sclerosis	7	4	3
Mixed cellularity	23	20	3
Others (LR and LD)	5	2	3

Abbreviations: LR, lymphocyte rich; LD, Lymphocyte depletion
