# Supplementary figures and images for: PD-1 and LAG-3 expression in EBV-associated pediatric Hodgkin lymphoma has influence on survival
Source: Front Oncol. 2022 Aug 5;12:957208. doi: 10.3389/fonc.2022.957208 (PMC9390066; doi:10.3389/fonc.2022.957208)

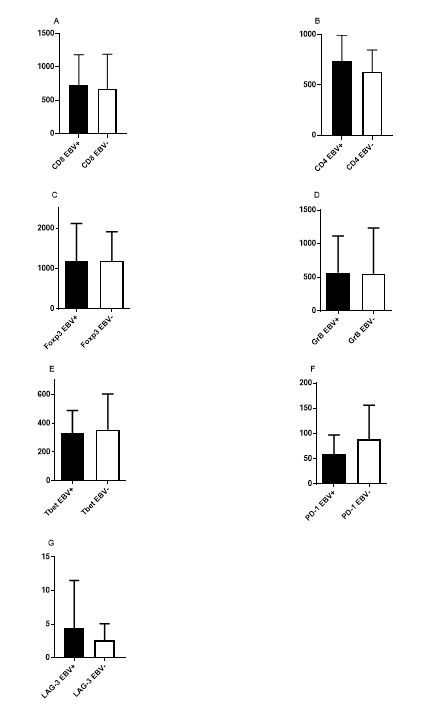

Supplement: Supplementary Figure 1 — Comparison of mean cell count between EBV+ and EBV- cases for (A) CD8, (B) CD4, (C) Foxp3, (D) GrB, (E) Tbet, (F) PD-1, (G) LAG-3. [file Image_1.tif]

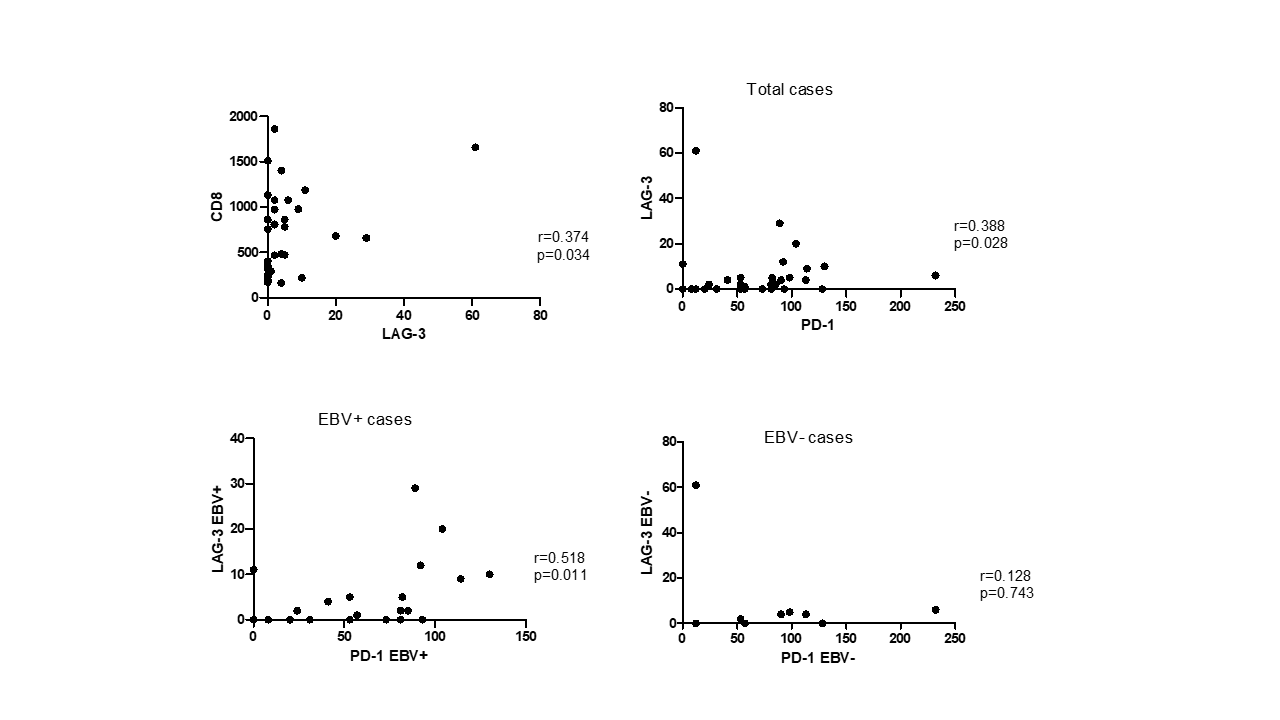

Supplement: Supplementary Figure 2 — Correlation analysis for LAG-3 with CD8 in total cases, and between PD-1 and LAG-3 in total cases, EBV+ and EBV- cases. [file Image_2.tif]
